# Nutraceuticals in Immune Function

**DOI:** 10.3390/molecules26175310

**Published:** 2021-09-01

**Authors:** Soo-Liang Ooi, Sok-Cheon Pak

**Affiliations:** School of Dentistry and Medical Sciences, Charles Sturt University, Bathurst, NSW 2795, Australia; sooi@csu.edu.au

Nutraceutical, a term derived from ‘nutrition’ and ‘pharmaceutical’, refers to any product isolated from herbs, nutrients, specific diets, processed foods, and beverages used not only for nutritional but also for medicinal purposes. Nutraceuticals comprise many bioactive derivatives from edible sources such as anti-oxidants, phytochemicals, fatty acids, amino acids, prebiotics and probiotics. Many of them possess therapeutic properties that can affect the immune system. In this Special Issue, AlAli et al. [1] offer a comprehensive review of nutraceuticals categorized based on their source, nature and application. The authors broadly classified the mode of actions of nutraceuticals into anti-cancer, anti-inflammatory, anti-oxidant, and anti-lipid activities. Nutraceuticals can augment the immune system to prevent cancer, neurological conditions, gastroenterological disorders, inflammatory diseases, and infections.

**Cancer:** Henamayee et al. [2] reviewed the anti-cancer properties of rhein, a naturally derived anthraquinone found in the rhubarb (*Rheum rhabarbarum*) leaves and many *Aloe* species. Current research supports rhein to be a multitargeted cytotoxicity compound. It affects several pathways such as mitogen-activated protein kinase (MAPK), Wnt, nuclear factor-kappa B (NF-κB) and hypoxia inducible factor-1 signaling to stimulate apoptosis and inhibit cell proliferation and angiogenesis. The chemopreventive activity of rhein was demonstrated in vivo or in vitro with various cancer types, including breast, cervical, colon, glioma, leukemia, liver, lung, nasopharyngeal, ovarian, pancreatic, and oral.

Anti-cancer cytotoxicity can directly or indirectly restore the suppressed immune response within the tumor microenvironment. Ooi et al. [3] reviewed the health-promoting properties and clinical applications of rice bran arabinoxylan compound (RBAC), a hydrolyzed extract of defatted rice bran modified with the shiitake mushroom enzyme. RBAC is also an anti-cancer neutraceutical that can upregulate the cytotoxic activity of natural killer cells, enhance phagocytic cellular functions, and induce the maturation and activation of dendritic cells. Moreover, the immunomodulatory, anti-inflammatory, anti-oxidant, and anti-angiogenic properties of RBAC are also promising for a wide range of applications beyond cancer.

Thymol is a phenolic compound found in the essential oil of thyme (*Thymus spp.*). Günes-Bayir et al. [4] studied the cytotoxic, genotoxic, and anti-oxidative effects of thymol on healthy cells and gastric adenocarcinoma cells. The study demonstrated that thymol at low concentrations provides anti-oxidative protection to healthy cells in vitro while inducing toxic effects in adenocarcinoma cells. The dose-dependent hormetic impact of thymol of different cell lines makes thymol a potential anti-cancer agent.

In another in vitro experiment, Panahipour et al. [5] showed that micellar casein and whey powder maintain the transforming growth factor-β (TGF-β) activity and its capacity to regulate the inhibitor of deoxyribonucleic acid binding (ID) 1 and ID3 genes in oral fibroblasts and oral squamous carcinoma cells, respectively. While the TGF-β activity and over-expression of the ID3 gene are known to link to oral cancer, the potential application of casein and whey powder in its prevention needs further research.

**Neurological conditions:** Ginseng (*Panax spp.*) is a widely used immune modulator in the traditional medicine of East Asia. Ginseng is also known to protect against neurodegeneration and neuroinflammation. Calabrese [6] reviewed the available literature on the hormetic dose-response effects of ginseng and its constituents (ginsenosides Rg1, Rb1, Rc, Rd, Re, ginseng saponins, gintonin, polyacetylenes). The author found evidence supporting the generality of such effects, especially in the neuroprotective studies of Parkinson’s disease, Alzheimer’s disease, stroke, and neonatal brain hypoxia. Hormesis is characterized by low-dose stimulation and high-dose inhibition. Hence, due to its popularity, overconsumption of ginseng in the population can be a public health concern.

Chou et al. [7] studied the effects of glucosamine in brain cognitive performance with an in vivo model. Glucosamine is an amino sugar and a prominent precursor in the biochemical synthesis of glycosylated proteins and lipids. The study found evidence of glucosamine exerting a cognition-enhancing function in the experimental mice through upregulating the brain-derived neurotrophic factor (BDNF) levels via the dependency pathway of cyclic adenosine 3′,5′-monophosphate (cAMP), protein kinase A, and cAMP response element-binding protein. As abnormal BDNF levels might be due to the chronic inflammatory state of the brain, glucosamine may also have applications in neuroinflammatory disorders such as Alzheimer’s disease, Parkinson’s disease, fibromyalgia, multiple sclerosis and chronic pain.

Peripheral nerve injuries (PNI) can also induce neuroinflammation. Vitamin B complex was explored as a potential treatment by Ehmedah et al. [8] with a femoral nerve injury rat model. Treatment with B vitamins appeared to enhance the M1-to M2-macrophage polarization and accelerate the transition from the non-myelin to myelin-forming Schwann cells. Hence, B vitamins could potentially promote nerve repair through PNI-triggered processes of neuroinflammation and neurodegeneration.

**Gastroenterological disorders:** Uranga et al. [9] reviewed the effects of mast cells on irritable bowel syndrome (IBS), a disorder of the gut–brain axis. There is a close interaction between the immune system and the nervous system with mast cells playing a key mediation role. A variety of food components were found to affect the modulation of mast cell activity in a specific manner. These nutrient-derived bioactive compounds include fatty acids, lipid molecules, fat-soluble vitamins (D3 and E), amino acids (arginine, glutamine and glycine), carotenoids, polyphenolic compounds, and spices. They can reduce mast cell degranulation that is responsible for the de novo synthesis of mediators of the neuro-immune-endocrine alterations present in IBS.

López-Gómez et al. [10] reviewed the effects of nutraceuticals as modulators of enteric glial cells (EGC). Various compounds, particularly those with anti-oxidant activity, including L-glutamine, L-glutathione, quercetin, resveratrol, and palmitoylethanolamide, were found to exert local or systemic neuroprotective effects on the enteric nervous system. Hence, nutraceuticals targeting the EGCs can potentially prevent or reduce gastroenterological disorders.

**Inflammatory diseases:** Resveratrol is a natural phytoalexin polyphenol predominantly found in berries and grapes. Meng et al. [11] reviewed its anti-inflammatory actions and mechanisms. Resveratrol appeared to regulate inflammatory response through various signaling pathways, including the arachidonic acid, NF-κB, MAPK, activator protein (AP)-1 transcription factor, and anti-oxidant defense pathways. Hence, there exist multiple lines of compelling evidence that resveratrol can play a promising role in managing autoimmune and inflammatory chronic diseases. One such condition is Duchenne muscular dystrophy (DMD), a progressive and fatal neuromuscular disorder with no cure. Woodman et al. [12] treated mdx mice with a low dose of resveratrol (5 mg/kg body weight/day) for 15 weeks. The study found resveratrol to reduce exercise-induced muscle necrosis in dystrophic muscle and lower gene expression of immune cell markers cluster of differentiation (CD) 86 and CD163. Nevertheless, signaling targets associated with resveratrol’s mechanism of action, including Sirtuin 1 and NF-κB, were unchanged. This study confirmed that resveratrol could be a therapeutic candidate for DMD treatment.

Astaxanthin is another nutraceutical compound with potent anti-inflammatory properties. It is a lipid-soluble, red-orange carotenoid accumulated in many marine creatures, such as lobsters, shrimp, trout, and salmon. Chang and Xiong [13] reviewed the anti-inflammatory mechanisms of astaxanthin. Astaxanthin was found to attenuate many inflammatory biomarkers through multiple signaling pathways, including phosphatidylinositol-3-kinase/protein kinase B (Akt), nuclear factor erythroid 2-like 2, NF-κB, extracellular-signal-regulated kinase, c-Jun N-terminal kinases, p38 MAPK, and the Janus kinase 2/signal transducer and activator of transcription 3. Moreover, astaxanthin was confirmed experimentally to alleviate chronic and acute inflammation in various diseases, such as neurodegenerative disorders, diabetes, gastrointestinal disease, renal inflammation, as well as skin and eye diseases.

Atherosclerosis is characterized by low-grade, chronic inflammation of the arterial wall. The review by Eshghjoo et al. [14] showed that many microbiota-derived metabolites were associated with atherosclerosis. For example, trimethylamine-N-oxide, a by-product of gut microbial metabolism of L-carnitine and choline after ingestion of eggs, meat, or fish, can elevate oxidized low-density lipoprotein and increased plaque formation. Accumulation of indoxyl sulphate, a metabolite converted from dietary tryptophan, can cause coronary calcification leading to atherosclerosis. Whereas indole, another gut microbiota-derived tryptophan catabolite, is an agonist for the aryl hydrocarbon receptor with anti-inflammatory effects that can prevent atherosclerosis. Hence, indole can be a promising nutraceutical for cardiovascular disease prevention.

Two studies investigated the role of different nutraceuticals in suppressing airway inflammation. In the first study, Shin et al. [15] studied how Korean red ginseng (KRG) could prevent airway inflammation triggered by Asian sand dust (ASD). KRG and its active compound ginsenoside Rg3 significantly suppressed ASD-induced NF-κB expression and activity. Furthermore, KRG and Rg3 inhibited ASD-induced mucin gene expression and protein production from bronchial epithelial cells in vitro. In another study, Choi et al. [16] demonstrated that the anti-oxidant lycopene could inhibit cytokine expression induced by house dust mites. Lycopene, a naturally occurring chemical that gives fruits and vegetables a red color, possibly suppressed the activation of toll-like receptor 4 and reduced the intracellular and mitochondrial oxidative stress in respiratory epithelial cells. Interferonopathies are monogenic autoinflammatory diseases characterized by disturbance of interferon-mediated immune responses. Genova et al. [17] showed that sulforaphane, a bioactive molecule in cruciferous vegetables, could modulate the stimulator of interferon genes (STING) mediated inflammation and interferon-stimulated genes expression in vitro. However, the study could only reproduce a trend towards the downregulation of STING in vivo. Further in vivo research is needed to confirm the findings.

**Infectious pathogens:***Helicobacter pylori* infection can lead to gastric inflammation, ulcers, and gastric cancer progression. Bae et al. [18] confirmed β-carotene as a potential treatment to prevent *H. pylori*-induced inflammation. β-carotene is the red-orange pigment abundant in fungi, plants, and fruits. It was shown in vitro to inhibit the *H. pylori*-induced activation of MAPKs and AP-1, expression of matrix metalloproteinase-10, and cell invasion. Moreover, β-carotene promoted the expression of peroxisome proliferator-activated receptor-gamma and catalase, which reduced oxidative stress in *H. pylori*-infected cells.

Coronavirus disease 2019 (COVID-19) is currently affecting the world ferociously with multiple waves of infections and variants. Bae and Kim [19] reviewed the literature on the potentially beneficial roles of vitamin C, D, and selenium for COVID-19. Vitamin D improves the physical barrier against viruses and stimulates the production of antimicrobial peptides. Selenium enhances the function of cytotoxic effector cells, whereas vitamin C is considered an anti-viral and anti-inflammatory agent as it increases immunity. For these reasons, supplementing vitamin C, D, and selenium for COVID-19 patients may help to boost the immune system, prevent virus spread, and reduce the disease progression.

In conclusion, how natural foods and nutritional products can improve health and immunity beyond their nutritional values is a phenomenon of interest in current research. This Special Issue brings together 19 scholarly articles exploring various neutraceuticals in immune function, through reviews or experiments against cancer, neurological conditions, gastroenterological disorders, inflammatory diseases, and infections. The findings are promising. While most nutraceuticals are generally safe for consumption, many also demonstrated hormetic dose-response effects. Hence, more studies on the safety and toxicities of nutraceuticals are needed to advise on their effective utilization.

## Abbreviations

The following abbreviations are used in this manuscript:

AP	activator protein
ASD	Asian sand dust
cAMP	cyclic adenosine 3′,5′-monophosphate
CD	cluster of differentiation
COVID-19	Coronavirus disease 2019
DMD	Duchenne muscular dystrophy
EGC	enteric glial cells
IBS	irritable bowel syndrome
ID	inhibitor of deoxyribonucleic acid binding
KRG	Korean red ginseng
MAPK	mitogen-activated protein kinase
NF-κB	nuclear factor-kappa B
PNI	peripheral nerve injuries
RBAC	rice bran arabinoxylan compound
STING	stimulator of interferon genes
TGF-β	transforming growth factor beta
